# Signal-to-Noise Ratio Enhancement of Single-Voxel In Vivo ^31^P and ^1^H Magnetic Resonance Spectroscopy in Mice Brain Data Using Low-Rank Denoising

**DOI:** 10.3390/metabo12121191

**Published:** 2022-11-29

**Authors:** Yeong-Jae Jeon, Shin-Eui Park, Keun-A Chang, Hyeon-Man Baek

**Affiliations:** 1 Department of Health Sciences and Technology, Gachon Advanced Institute for Health Sciences and Technology, Gachon University, Incheon 21999, Republic of Korea; 2 Department of Biomedical Science, Lee Gil Ya Cancer and Diabetes Institute, Gachon University, Incheon 21999, Republic of Korea; 3 Department of Basic Neuroscience, Neuroscience Research Institute, Gachon University, Incheon 21999, Republic of Korea; 4 Department of Molecular Medicine, Lee Gil Ya Cancer and Diabetes Institute, Gachon University, Incheon 21999, Republic of Korea

**Keywords:** single voxel, ^31^P MRS, ^1^H MRS, denoising, mouse brain, stroke

## Abstract

Magnetic resonance spectroscopy (MRS) is a noninvasive technique for measuring metabolite concentration. It can be used for preclinical small animal brain studies using rodents to provide information about neurodegenerative diseases and metabolic disorders. However, data acquisition from small volumes in a limited scan time is technically challenging due to its inherently low sensitivity. To mitigate this problem, this study investigated the feasibility of a low-rank denoising method in enhancing the quality of single voxel multinuclei (^31^P and ^1^H) MRS data at 9.4 T. Performance was evaluated using in vivo MRS data from a normal mouse brain (^31^P and ^1^H) and stroke mouse model (^1^H) by comparison with signal-to-noise ratios (SNRs), Cramer-Rao lower bounds (CRLBs), and metabolite concentrations of a linear combination of model analysis results. In ^31^P MRS data, low-rank denoising resulted in improved SNRs and reduced metabolite quantification uncertainty compared with the original data. In ^1^H MRS data, the method also improved the SNRs, CRLBs, but it performed better for ^31^P MRS data with relatively simpler patterns compared to the ^1^H MRS data. Therefore, we suggest that the low-rank denoising method can improve spectra SNR and metabolite quantification uncertainty in single-voxel in vivo ^31^P and ^1^H MRS data, and it might be more effective for ^31^P MRS data. The main contribution of this study is that we demonstrated the effectiveness of the low-rank denoising method on small-volume single-voxel MRS data. We anticipate that our results will be useful for the precise quantification of low-concentration metabolites, further reducing data acquisition voxel size, and scan time in preclinical MRS studies.

## 1. Introduction

Phosphorous and proton magnetic resonance spectroscopy (^31^P and ^1^H MRS) are noninvasive techniques that have been used to study metabolic changes in the brains of small animals [1,2]. Data acquisition of low-concentration metabolites from small brain volumes are technically challenging because of the insufficient signal-to-noise ratio (SNR) in the limited scan time. The signal-to-noise ratio can be improved by increasing the number of signals averaged; therefore, it is impractical, especially for ^31^P MRS or disease animal model studies (e.g., stroke model [3,4,5,6]), because of the increased scan times that frequently result in sudden animal death and motion artifacts.

To improve the SNR and thereby reduce the uncertainty of the ^31^P and ^1^H MRS data analyses, there is an approach for reducing noise in the data by postprocessing. One of the simplest methods is apodization in the time domain to smooth the data, which results in line broadening, thus, decreasing the spectral resolution [7]. To mitigate this problem, several noise reduction methods have also been proposed for decomposing signals, such as low-rank [8,9], Fourier thresholding [10,11], and principal component analysis [12,13] methods. Although these methods have been applied to ^31^P and ^1^H MRS data, most are aimed at human studies using 3 T or 7 T and chemical shift imaging data. It seems that there are a few studies that apply denoising methods to animal data at 9.4 T and single voxel in vivo MRS data.

Ahmed [10] reported that the wavelet shrinkage denoising method for single-voxel in vivo ^31^P MRS human brain data could enhance the SNR from 4.9 dB before processing to 17.7 dB after processing. However, the in vivo ^31^P spectrum displayed in this study is a magnitude spectrum, and some metabolites have not resolved. It is unclear whether this method can reduce uncertainty and preserve the metabolite information for fitting analysis. On the other hand, Rowland et al. [11] studied the performance of spectral improvement using the Fourier thresholding (SIFT) denoising method for in vivo dynamic ^31^P MRS muscle data. They reported that the SIFT method considerably improved SNR and substantially reduced the uncertainty of spectral fitting; however, their method was not reported to be effective for ^31^P and ^1^H MRS mouse brain data. Therefore, we considered that the wavelet shrinkage method might also be very effective for single voxel ^31^P and ^1^H MRS animal brain data at 9.4 T.

A previous study reported that the low-rank denoising method was successful for ^13^C MRS animal data [14]. They reported that, singular value decomposition (SVD)-based low-rank denoising gives an order-of-magnitude improvement in SNR in DNP ^13^C tracer experiments, previously undetectable peaks can be quantified by low-rank denoising, higher SNR translates to more precise kinetic fitting, and dynamic single-voxel spectroscopy of glucose metabolism without DNP is possible. Therefore, we speculated that the low-rank denoising method might also be very effective for single-voxel ^31^P and ^1^H MRS animal brain data at 9.4 T. Finally, Clarke and Chiew thoroughly investigated the uncertainty in denoising using low-rank methods for ^1^H MRS human brain 3T data [15]. Denoising was carried out using the MRS-denoising-tools Python package and a fitting tool [16]. They reported that low-rank denoising methods based on spatiotemporal or dynamic temporal separability reduced the uncertainty in ^1^H MRS or ^1^H chemical shift imaging data, but linear predictability [17] for single-voxel ^1^H MRS was not effective at 3 T. However, it might be necessary to investigate more for single-voxel ^31^P and ^1^H MRS data at 9.4 T.

In this study, we investigated the noise reduction effects of the low-rank denoising methods by utilizing the linear predictability implemented in the MRS-denoising-tools Python package [16]. We applied this method to various mouse-brain MR spectra. We compared ^31^P MRS data acquired from normal mice with ^1^H MRS data acquired from normal and stroke mouse models. We performed a linear combination of model (LCModel) [18] analysis and compared estimated SNRs, Cramer-Rao lower bounds (CRLBs), and metabolite concentrations between raw and denoised data.

## 2. Materials and Methods

### 2.1. Low-Rank Denoising

**Figure 1:**
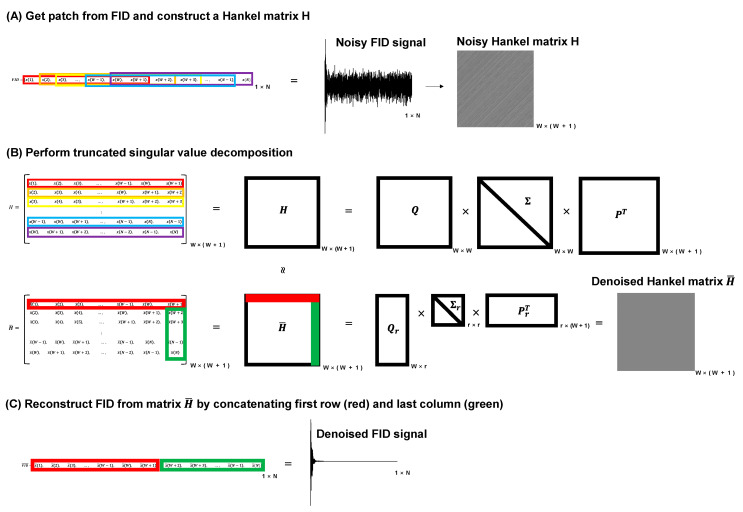
Schematic representation of the low-rank denoising approach. (**A**) Conversion of the FID patch signal to a Hankel matrix, H. (**B**) Truncated SVD of the Hankel matrix, H. (**C**) Reconstruction of the noise-reduced FID using the denoised Hankel matrix, $\bar{H}$. SVD, singular value decomposition; N, number of data points; r, rank or number of basis spectra; W = N/2; H, Hankel matrix; FID: free induction decay signal; $\bar{H}$, denoised Hankel matrix; $\bar{FID}$, denoised FID signal.

The low-rank denoising approach, which is based on previous studies, is shown in Figure 1 [12,15]. This method utilizes the low-rank structure of a Hankel matrix constructed using single-voxel time-domain free induction decay (FID) data (Figure 1A). The matrix size (W × W + 1) is typically determined by default as the half-length (N/2 = W) of the signal. After constructing the Hankel matrix H, SVD is applied with a rank parameter, r (Figure 1B), and the denoised FID signal is reconstructed by concatenating the first row and last column of the denoised Hankel matrix, $\bar{H}$ (Figure 1C). Noise reduction is achieved by reducing the rank parameter of the Hankel matrix, which is usually selected as the number of independent metabolite peaks or basis functions. In this study, we used W as the half-length of the signal and r as the number of metabolites (e.g., r = 13 for ^31^P MRS, and r = 16 for ^1^H MRS, see Figures S1–S4). Line broadening of 5–15 Hz was applied before applying the low-rank denoising for ^31^P MRS data because the denoising method did not work for some very noisy raw data. The method is implemented in Python with an Intel Core i7-12700KF 3.61 GHz CPU and 32-GB memory. The average execution times of denoising on the ^31^P and ^1^H datasets were 5.58 s and 0.88 s, respectively.

### 2.2. Animals

For ^31^P and ^1^H MRS experiments, wild-type male C57BL/6N mice (N = 13, ^31^P MRS, and N = 13, ^1^H MRS) were obtained from ORIENT BIO Inc. (Seongnam, Republic of Korea) and studied at 8 weeks of age. The mice, which had unconstrained access to water and food, were housed in the Center of Animal Care and Use (CACU), where consistent conditions were maintained at a certain temperature (21–23 °C) and humidity (50–60%), with a 12-h light/dark cycle. The study was approved by the Institutional Animal Care and Use Committee of the Lee Gil Ya Cancer and Diabetes Institutional Center, which abides by the guidelines of the Institute of Laboratory Animal Resources. For the ^1^H MRS experiments, stroke mice (N = 3) were modeled using the standard method [19,20].

### 2.3. General Set-Up for MR Experiments

All experiments were carried out on a BioSpec 9.4 T MRI system with ParaVision 6.0 software (Bruker BioSpin Corporation, Billerica, MA, USA) at the Core-Facility for Cell to In Vivo Imaging. During all the MR experiments, the mice were positioned supine on a cradle and their brains were scanned. The mice were anesthetized by spontaneous inhalation of 2.0–2.5% isoflurane, a 1:2 mixture of O_2_ and NO_2_ (250 mL/min), using a nose cone and anesthesia unit placed on the cradle. Anesthesia was maintained according to respiratory rate, and respiration was monitored during all MRI/MRS procedures. A circulating water heating system was used to keep the mice warm during the scans.

### 2.4. ^31^P MRS Acquisition

The ^31^P MRS data were acquired at 9.4 T using a dual-tuned ^1^H/^31^P surface coil of 20 mm with an Image Selected In Vivo Spectroscopy (ISIS) [21] pulse sequence, consisting of three frequency-selective inversion pulses (5.19 ms; 16,000 Hz) and an excitation pulse (0.08 ms, 16,000 Hz, and 20 W). The acquisition parameters had a repetition time (TR) of 4000 ms, 128 ISIS averages, 4096 complex data points, and 16,025.64 Hz spectral bandwidth.

For the ^31^P MRS in the mouse brain, a single voxel was in the whole brain region (6.0 × 4.0 × 6.0 mm^3^). After careful localization of the voxel, localized shimming was initiated using the localized shimming scan. The local reference frequency was set to that of the dominant resonance of the ^1^H signal in the selected voxel using the local frequency method, and the first-order shims were automatically adjusted based on the unsuppressed water signal in the selected voxel using the local shim method, and then the local frequency was readjusted according to the adjusted shims. The full width at half maximum (FWHM) of the unsuppressed water signal was used as a reference for the localized shimming performance.

### 2.5. ^1^H MRS Acquisition

For the ^1^H MRS acquisition, a quadrature volume resonator was used for signal excitation, and a four-channel array coil was used for signal reception. A violet-colored box (VOI) was placed in the mouse brain near the striatum region and in a stroke lesion. A localized shim scan was performed to improve field homogeneity in the VOI. After the shimming, ^1^H MRS data were acquired using a point-resolved spectroscopy pulse sequence [22] with the following acquisition parameters: TR = 5000 ms, TE = 17.54 ms, 320 averages, data points = 2048, spectral width = 4401.41 Hz, and VOI size = 2 × 2 × 2 mm^3^. Variable pulse power and optimized relaxation delays [23] were used to suppress the water signal, and outer volume suppression was performed to suppress unwanted signals outside the VOI.

### 2.6. ^1^H MRI Acquisition

Multislice T2-weighted images were obtained to provide a geometrical reference for locating voxels. A T2 turbo-rapid-acquisition-with-relaxation enhancement (RARE) sequence [24,25] and the following parameters were used to obtain axial and coronal MRI in the brains of the mice: TR = 3200 ms; TE = 11 ms; effective TE = 33 ms; RARE factor = 8, averages = 4; field of view = 15 × 15 mm^2^; matrix size = 150 × 150; and slice thickness = 0.50 mm.

### 2.7. LCModel Analysis

All the ^31^P and ^1^H MRS data were analyzed using LCModel with prior knowledge. To analyze the ^31^P spectra, a simulated basis set was generated using the Spinach toolbox [26], assuming the pulse-acquire sequence using reported chemical shifts and J-coupling constant information [27]. The basis set consisted of 13 basis spectra (PCr, α-ATP, β-ATP, γ-ATP, Pi, NADH, NAD^+^, PE, PC, GPE, GPC, MP, and DPG). For the ^31^P fitting using the LCModel, the following input parameters were used, as in Deelchand et al. [27]: DKNTMN = 2 × 99, XSTEP = 5, RFWHM = 3, FWHMBA = 0.049, NREFPK(2) = 1, PPMREF(1,2) = 0, DESDSH = 0.01, ALPBMN = 7.8 × 10^−10^, and ALPBMX = 3.9 × 10^−7^. The LCModel fitting was conducted over the spectral range of −19.5–10 ppm. To analyze the ^1^H spectra using LCModel, we used a vendor-provided basis set based on the chemical shifts and J-coupling constant information [28]. The following metabolites were included in the basis set: alanine (Ala), aspartate (Asp), creatine (Cr), phosphocreatine (PCr), GABA, glucose (Glc), glutamine (Gln), glutamate (Glu), GPC, PCh, GSH, Ins, Lac, NAA, NAAG, and Tau. The LCModel fitting was conducted over a spectral range of 0.2–4.0 ppm. The SNR is defined here as the ratio of the maximum in the spectrum minus the baseline over the analysis window to twice the rms residuals. The CRLBs are standard error estimates, or the estimated standard deviations of the estimated concentrations expressed in percent %SD in LCModel.

### 2.8. Statistical Analysis

Statistical analyses were performed using MATLAB. The mean SNR, CRLB, and concentration values from the 31P and 1H MRS data were compared between the unsmoothed raw spectra and the denoised spectra using paired t-tests. Statistical significance was set at *p* < 0.05. We removed the not detected data (e.g., %SD = 999) in the LCModel output for the statistical analysis.

## 3. Results

### 3.1. Normal Brain ^31^P MRS

**Figure 2:**
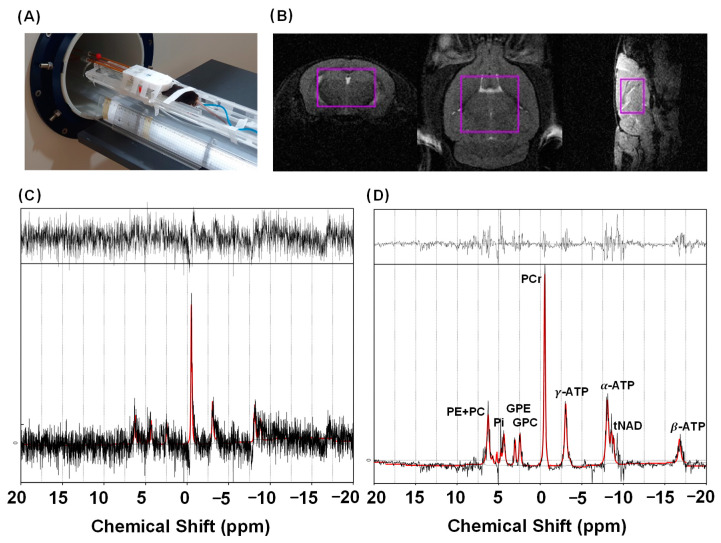
^31^P MRS data acquisition and analysis results. Mouse positioning (**A**), ^31^P MRS voxel positioning of the inner brain region (violet color box) (**B**), LCModel analysis without denoising (**C**), and LCModel analysis with denoising (**D**). Note: in (**C**,**D**), the red lines represent LCModel spectral fitting results, and the black lines are target in vivo ^31^P spectra (bottom) and residual signals (top).

The aim of the first in vivo study was to examine the performance of the low-rank denoising applied to a normal ^31^P MRS data set. The mouse positioning (Figure 2A), data acquisition VOI (Figure 2B, violet color box), and LCModel analysis results before (Figure 2C) and after (Figure 2D) the denoising are shown in Figure 2. The low-rank denoising resulted in a dramatically increased mean SNR from 5.08 to 15.62, or 207.76% (Table 1, *p* < 0.001). Table 2 shows the CRLBs and metabolite concentration ratios for normal 31P MRS data. The low-rank denoising resulted in lower CRLB values for PCr, α-ATP, β-ATP γ-ATP, GPC, Pi, PC, PE, DPG, NAD+, and NADH (*p* < 0.05), and there were statistically significant differences in metabolite concentration ratios for α-ATP, β-ATP, γ-ATP, GPC, Pi, PC, and PE.

### 3.2. Normal Brain ^1^H MRS

**Figure 3:**
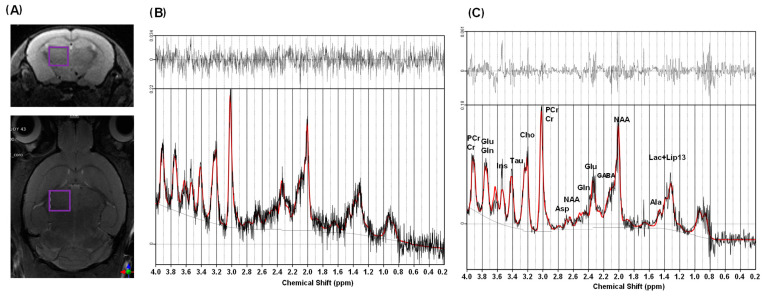
^1^H MRS data acquisition and analysis results. ^1^H MRS voxel positioning on striatum (violet-colored box) (**A**), LCModel analysis without denoising (**B**), and LCModel analysis with denoising (**C**). Note: in (**B,C**), the red lines represent LCModel spectral fitting results, and the black lines are target in vivo ^1^H spectra (**bottom**) and residual signals (**top**).

For the second in vivo study, normal ^1^H MRS data (Figure 3) were used to evaluate the low-rank denoising performance. The low-rank denoising was applied to the normal ^1^H MRS data, and then its LCModel analysis results were compared to the original unsmoothed data. Figure 3A presents the VOI for normal ^1^H MRS data, and Figure 3B,C show the LCModel analysis results with unsmoothed and denoised normal ^1^H MRS data, respectively. The mean and standard deviation (SD) of the SNR were calculated for the VOI. The results are presented in Table 1 and indicate that low-rank denoising increased the mean SNR from 71.12 to 91.41, or 28.57% (Table 1, *p* < 0.001). Table 3 presents the CRLBs and metabolite concentrations for normal ^1^H MRS data. The low-rank denoising resulted in lower CRLB values for PCr, Glc, GSH, NAA, Tau, tCho, tNAA, tCr, and Glx (*p* < 0.05), and there were statistically significant concentration differences for PCr, GABA, Glc, Gln, Glu, GSH, NAA, NAAG, Tau, tNAA, and tCr (*p* < 0.05).

### 3.3. Stroke Lesion ^1^H MRS

**Figure 4:**
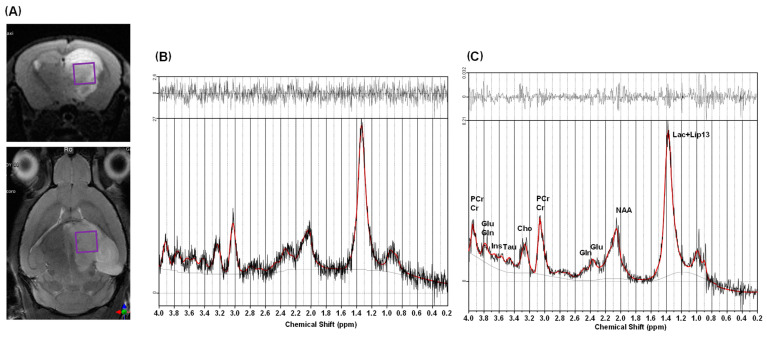
^1^H MRS data acquisition and analysis results. ^1^H MRS voxel positioning on stroke lesion (violet-colored box) (**A**), LCModel analysis without denoising (**B**), and LCModel analysis with denoising (**C**). Note: in (**B**,**C**), the red lines represent LCModel spectral fitting results, and the black lines are target in vivo ^1^H spectra (**bottom**) and residual signals (**top**). Glx is Glu + Gln.

For the third in vivo study, “stroke” ^1^H MRS data (Figure 4) were used to evaluate low-rank denoising performance. The low-rank denoising was applied to the “stroke” ^1^H MRS data, and then its LCModel analysis results were compared to the original unsmoothed data. Figure 4A presents the VOI for “stroke” ^1^H MRS data and Figure 4B,C show LCModel analysis results with unsmoothed and denoised “stroke” ^1^H MRS data, respectively. The mean and SD of the SNR were calculated for the VOI. The results are presented in Table 1, which show that the low-rank denoising increased the mean SNR from 10.35 to 13.67 and 36.67% (Table 1, *p* = 0.0533). Table 4 presents the CRLBs and metabolite concentrations for “stroke” ^1^H MRS data. Low-rank denoising resulted in a lower CRLB value only for tCho (*p* < 0.05), and there were no statistically significant differences for other CRLBs or all metabolite concentrations.

## 4. Discussion

In this study, we utilized multinucleus in vivo MRS data obtained from mouse brains to investigate the noise reduction performance of the low-rank denoising method using LCModel analysis. We analyzed not only in vivo ^31^P MRS data from normal mouse brains but also in vivo ^1^H MRS data from normal and stroke lesions. The main finding of this study is that the low-rank denoising method can significantly improve the SNR and reduce the LCModel fitting uncertainty in single-voxel multinuclei 9.4 T MRS data without significant loss of metabolite information.

Using the ^31^P MRS data, it was shown that the low-rank denoising resulted in a higher SNR and lower CRLB values compared to the original data for all the metabolites. For PCr, α-ATP, γ-ATP, and PE, the CRLB values decreased, but the concentration ratios increased. One reason seems to be that proper spectral phase correction failed when the LCModel analysis of the original data was performed (Figure 2C). As seen from the real spectrum in Figure 2C, phases, such as PCr, α-ATP, and β-ATP were not corrected properly compared to the denoised spectrum (Figure 2D). Therefore, the concentration ratios of the original data were underestimated, and the denoised data were more accurate. For DPG, NAD^+^, and NADH, the CRLB values were reduced, and there were no statistically significant differences in the concentration ratios, indicating that metabolite information was preserved after denoising. Therefore, these results show that low concentrations of metabolites can be reliably detected in single voxel 9.4 T ^31^P MRS data. Cerebral NAD content plays an important role in the brain and is known to contribute to various neurological diseases [29,30,31]. Skupienski et al. [29] reported that NAD^+^ and NADH were quantified using the LCModel with a CRLB of 10% and 14%, respectively, at 14.1 T. This result is comparable to our data. Therefore, we believe that the low-rank denoising method might be useful for studying neurodegenerative disorders using ^31^P MRS at 9.4 T.

Using normal ^1^H MRS data, the results indicated good performance in reducing the uncertainty for some prominent metabolites (e.g., PCr, Glu, GSH, NAA, Tau, tCho, tNAA, tCr, and Glx). However, there were no improvements or differences in uncertainty for some metabolites having complex multiplet signals (e.g., Ala, Asp, GABA, Glc, Lac, and NAAG), and we observed even increased CRLBs (e.g., Glc and Glx) with denoising. It might be due to artifact peaks (green arrows in Figure S2) for Glx and a loss of signals (violet arrows in Figure S2) for Glc. There were also statistically significant differences in metabolite concentrations for PCr, Glc, Glu, GSH, NAA, Tau, tNAA, tCr, GABA, Gln, and NAAG after denoising compared to the original data. We interpret that these results show that applying the low-rank denoising method to single-voxel 9.4 T ^1^H MRS data resulted in the loss of metabolite information. According to Clarke et al. [15], it was reported that this low-rank denoising method, called linear predictability denoising for single-voxel MRS data, did not decrease uncertainty and was not effective when applied to ^1^H MRS data at 3 T, and it seems to apply to our 9.4 T ^1^H MRS data.

The results of the third in vivo ^1^H MRS study with a stroke lesion also indicated similar denoising performance to the normal ^1^H MRS data in terms of SNR and fitting uncertainty, but there were no significant differences between the original and denoised data except for tCr. The overestimation found in the healthy mice was not observed in the stroke model. The metabolite signals from the stroke brain are already severely degraded compared to the normal brain and it might be due to loss of signals due to denoising (violet arrows in Figure S3).

We expected improved detectability of the Lac peak after denoising, especially because it is known to increase in the stroke model [3,4,5,6]. However, we could not observe any evidence that low-rank denoising would improve Lac detection using LCModel analysis. Specifically, LCModel fitting was confused with Lac and lipid at 1.3 ppm. These results may be improved using the spectral registration technique [32,33] and LCModel fitting analysis with a customized basis set considering the shaped RF pulses [34,35,36]. It should also be mentioned that SNR enhancement techniques are important to protect against animal death, especially in disease models, by reducing scan times.

This study has several limitations. First, the size of the VOI for ^31^P MRS data acquisition was too large; it included almost the whole mouse brain, and not a single structural brain region. We need to demonstrate its feasibility with denoising on the mouse brain in further studies. Second, we observed common spurious peaks in the denoised spectra and irregular non-Gaussian noise patterns in the residual signals (Figure 2D, Figure 3C and Figure 4C), indicating that the low-rank denoising and LCModel fitting were not perfect. One possible method for mitigating this is to give the LCModel more flexibility to estimate the line broadening for each metabolite and use an “adjusted linewidth” basis set for LCModel fitting, as suggested by Deelchand et al. [27].

To further improve these problems, other methods might need to be considered in future studies, such as wavelet shrinkage [10], SIFT [11], deep learning-based denoising [37,38,39], low-rank tensor modeling [40,41], SNR-optimized Ernst angle acquisition [42], and using more advanced pulse sequences, such as polarization transfer [43], fitting analysis with more realistic line shapes [27], and spectral registration [32,33].

## 5. Conclusions

In this study, we utilized a low-rank denoising technique to investigate the noise reduction performance of single-voxel in vivo ^31^P and ^1^H mouse brain spectra at 9.4 T. We found a significant SNR enhancement and improved the LCModel fitting performance. We also found that low-concentration metabolites in ^31^P MRS, such as NAD^+^, NADH, PE, β-ATP, GPC, Pi, and PC can be reliably quantified using LCModel after denoising. We suggest that enhanced SNR in ^31^P and ^1^H spectra from the mouse brain implies a smaller acquisition VOI size, reduced scan time, and improved detection of low concentration metabolites. Finally, the denoising method performed better in ^31^P MRS data than in ^1^H MRS data, which might be due to the different sparsities of the data. We believe that these results could be useful for single-voxel mouse brain MRS data analyses. However, further studies are necessary to confirm these findings.

## Figures and Tables

**Table 1:** SNR differences between raw and denoised data.

Method	Region	SNR (Raw)	SNR (Low-Rank)	%Diff From Raw	*p*-Value
^31^P MRS (N = 13)	Whole brain	5.08 ± 1.67	15.62 ± 4.08	207.76	**<0.001**
^1^H MRS (N = 10)	Striatum	71.12 ± 1.12 ^1^	91.41 ± 1.41	28.57	**<0.001**
^1^H MRS (N = 3)	Stroke lesion	10.35 ± 3.46	13.67 ± 4.93	35.67	0.0533

^1^ Average SNR values of the spectra (mean ± standard deviation). Significant differences were calculated using pairwise t-tests. Bolded values represent SNRs (e.g., PCr for ^31^P MRS and NAA for 1H MRS) with significant differences. SNR, signal to noise ratio.

**Table 2:** CRLB and concentration differences between raw and denoised ^31^P MRS data.

	CRLB (%SD)			Concentration Ratio (/PCr)		
Metabolite	Raw	Low-Rank	*p*-Value	Raw	Low-Rank	*p*-Value
PCr	4.69 ± 1.38	1.31 ± 0.48	**<0.001**	1.00 ± 0.00	1.00 ± 0.00	NaN
α-ATP	12.46 ± 6.19	2.62 ± 0.77	**<0.001**	0.33 ± 0.12	0.74 ± 0.21	**<0.001**
β-ATP	20.57 ± 15.71	6.15 ± 2.67	**0.0355**	0.15 ± 0.06	0.24 ± 0.07	**0.0131**
γ-ATP	14.00 ± 6.04	3.38 ± 0.51	**<0.001**	0.34 ± 0.06	0.55 ± 0.14	**<0.001**
GPC	29.62 ± 10.63	10.25 ± 11.38	**<0.001**	0.12 ± 0.04	0.15 ± 0.08	**0.0104**
GPE	88.73 ± 67.82	72.25 ± 121.33	0.7789	0.04 ± 0.04	0.05 ± 0.05	0.7542
Pi	20.62 ± 10.53	5.92 ± 1.08	**<0.001**	0.18 ± 0.05	0.26 ± 0.10	**0.0105**
PC	29.92 ± 8.50	18.77 ± 14.85	**0.0062**	0.10 ± 0.04	0.07 ± 0.04	**0.0106**
PE	19.15 ± 11.51	8.00 ± 12.08	**<0.001**	0.21 ± 0.09	0.32 ± 0.16	**<0.001**
MP	107.89 ± 55.39	96.00 ± 72.89	0.1646	0.03 ± 0.03	0.02 ± 0.02	0.1615
DPG	38.92 ± 16.34	22.08 ± 25.50	**0.0288**	0.08 ± 0.03	0.07 ± 0.03	0.2322
NAD+	42.17 ± 23.15	14.17 ± 5.06	**0.0012**	0.10 ± 0.07	0.11 ± 0.06	0.3254
NADH	27.69 ± 28.27	14.83 ± 7.08	**0.0295**	0.18 ± 0.06	0.14 ± 0.16	0.2927

Average CRLB and metabolite concentration ratios in the spectra (mean ± standard deviation). Significant differences were calculated using pairwise *t*-tests. Bolded values represent CRLB and concentration ratio values with significant differences. CRLB, Cramer-Rao lower bound; NaN, not available.

**Table 3:** CRLB and concentration differences between raw and denoised ^1^H MRS (normal) data.

	CRLB (%SD)			Concentration (mM/L)		
Metabolite	Raw	Low-Rank	*p*-Value	Raw	Low-Rank	*p*-Value
Ala	25.29 ± 10.03	17.38 ± 4.75	0.1105	1.82 ± 0.59	1.98 ± 0.54	0.5838
Asp	57.86 ± 24.14	55.17 ± 19.83	0.6064	1.33 ± 0.44	1.47 ± 0.61	0.5294
Cr	23.75 ± 7.17	33.86 ± 16.55	0.1042	3.94 ± 1.69	2.47 ± 0.63	0.0608
PCr	22.50 ± 14.47	10.13 ± 3.98	**0.0334**	5.14 ± 2.10	8.05 ± 1.94	**0.0116**
GABA	15.13 ± 2.85	16.50 ± 2.78	0.2111	2.91 ± 0.58	2.61 ± 0.40	**0.0359**
Glc	39.00 ± 15.54	88.38 ± 95.64	**0.0275**	1.28 ± 0.48	1.35 ± 0.97	**0.0074**
Gln	14.88 ± 3.94	16.13 ± 5.36	0.2168	3.57 ± 0.59	2.89 ± 0.59	**0.0021**
Glu	5.25 ± 0.46	4.00 ± 0.53	**0.0016**	9.58 ± 1.35	10.94 ± 1.60	**0.0241**
GPC	68.40 ± 28.69	69.00 ± 8.49	NaN	0.71 ± 0.51	0.55 ± 0.09	NaN
PCh	28.50 ± 25.88	10.38 ± 10.29	0.0673	1.19 ± 0.35	1.67 ± 0.47	0.0578
GSH	11.38 ± 1.30	8.50 ± 1.07	**0.0012**	2.38 ± 0.47	2.73 ± 0.43	**0.0021**
Ins	7.13 ± 1.64	7.00 ± 2.14	0.8264	5.53 ± 1.10	6.03 ± 1.63	0.2259
Lac	21.60 ± 7.83	9.75 ± 2.22	0.0572	2.71 ± 0.22	4.13 ± 0.82	0.0973
NAA	5.50 ± 0.93	3.75 ± 0.71	**<0.001**	6.74 ± 1.16	8.04 ± 1.47	**<0.001**
NAAG	25.75 ± 10.39	42.63 ± 49.77	0.2766	1.54 ± 0.51	1.12 ± 0.49	**0.0080**
Tau	5.38 ± 1.77	4.25 ± 0.71	**0.0379**	8.08 ± 2.24	9.32 ± 1.92	**0.0312**
tCho	5.75 ± 0.71	4.62 ± 0.74	**<0.001**	1.63 ± 0.36	1.81 ± 0.49	0.0552
tNAA	4.60 ± 0.53	3.63 ± 0.52	**0.0062**	8.28 ± 1.31	9.16 ± 1.60	**<0.001**
tCr	3.88 ± 0.64	3.00 ± 0.00	**0.0062**	9.08 ± 1.21	10.21 ± 1.35	**0.0154**
Glx	4.40 ± 0.76	4.50 ± 0.76	**0.0072**	13.15 ± 1.51	13.83 ± 1.85	0.2855

Average CRLB and metabolite concentration ratios in the spectra (mean ± standard deviation). Significant differences were calculated using pairwise t-tests. Bolded values represent CRLB and concentration ratio values with significant differences. CRLB, Cramer-Rao lower bound; NaN, not available.

**Table 4:** CRLB and concentration differences between raw and denoised ^1^H MRS (stroke) data.

	CRLB (%SD)			Concentration (mM/L)		
Metabolite	Raw	Low-Rank	*p*-Value	Raw	Low-Rank	*p*-Value
Ala	24.00 ± 17.58	24.67 ± 17.01	0.8685	1.91 ± 0.93	1.50 ± 0.74	0.1835
Asp	60.33 ± 26.76	55.33 ± 15.14	0.7785	0.97 ± 0.91	0.72 ± 0.48	0.4389
Cr	29.33 ± 30.83	23.67 ± 16.62	0.7715	3.11 ± 2.64	2.85 ± 2.57	0.5472
PCr	347.33 ± 564.46	346.33 ± 565.27	0.6784	2.27 ± 3.33	2.84 ± 4.46	0.4832
GABA	20.67 ± 3.79	28.67 ± 6.66	0.1689	1.34 ± 0.97	0.95 ± 0.51	0.3403
Glc	406.33 ± 517.88	417.67 ± 505.28	0.5532	0.14 ± 0.14	0.09 ± 0.08	0.4226
Gln	343.67 ± 567.54	21.33 ± 15.31	0.4185	1.92 ± 1.84	2.05 ± 1.75	0.2491
Glu	7.00 ± 1.73	6.67 ± 2.31	0.4226	5.52 ± 3.30	5.47 ± 4.12	0.9265
GPC	679.00 ± 554.26	358.00 ± 555.29	0.4136	0.05 ± 0.09	0.06 ± 0.07	0.6912
PCh	12.33 ± 7.51	34.67 ± 29.30	0.3470	1.01 ± 0.92	1.01 ± 0.94	0.6270
GSH	40.67 ± 20.09	24.67 ± 12.06	0.2513	0.88 ± 0.94	0.96 ± 1.08	0.4635
Ins	10.67 ± 4.04	10.67 ± 5.13	1.00	3.53 ± 3.01	3.73 ± 3.76	0.6787
Lac	30.00 ± 40.73	45.33 ± 66.40	0.4117	9.86 ± 8.76	9.77 ± 8.67	0.7703
NAA	10.00 ± 5.57	9.33 ± 5.51	0.4226	3.48 ± 3.56	3.87 ± 4.24	0.4275
NAAG	355.33 ± 557.43	368.00 ± 546.71	0.3755	0.41 ± 0.48	0.25 ± 0.27	0.3371
Tau	10.00 ± 6.08	9.00 ± 4.36	0.4226	5.30 ± 5.86	5.62 ± 6.54	0.4937
tCho	8.67 ± 3.51	7.00 ± 3.00	**0.0377**	1.06 ± 0.87	1.06 ± 0.91	0.9731
tNAA	10.00 ± 5.57	10.00 ± 5.57	NaN	3.89 ± 3.99	4.12 ± 4.47	0.4797
tCr	6.00 ± 3.61	5.33 ± 2.08	0.6349	5.39 ± 4.20	5.68 ± 4.63	0.4155
Glx	7.00 ± 1.73	7.00 ± 1.73	NaN	7.44 ± 5.10	7.52 ± 5.86	0.8728

Average CRLB and metabolite concentration ratios in the spectra (mean ± standard deviation). Significant differences were calculated using pairwise t-tests. Bolded values represent CRLB and concentration ratio values with significant differences. CRLB, Cramer-Rao lower bound; NaN, not available.

## Data Availability

Data are not shown due to the privacy.

## Supplementary Materials

The following are available online at https://www.mdpi.com/article/10.3390/metabo12121191/s1, Figure S1: The low-rank denoising results of in vivo ^31^P MRS normal mouse brain data with gradually increasing the number r. The blue lines are input noisy in vivo ^31^P spectra and orange lines are denoised spectra, r, rank; MSE, mean squared error. Figure S2: The low-rank denoising results of in vivo ^1^H MRS normal mouse brain data by gradually increasing the number r. The blue lines are input noisy in vivo ^1^H spectra and orange lines are denoised spectra. Zoomed spectra from the red box are shown, and artifact peaks and losses of signals are indicated with green and violet arrows, r, rank; MSE, mean squared error. Figure S3: The low-rank denoising results of in vivo ^1^H MRS stroke mouse brain data by gradually increasing the number r. The blue lines are input noisy in vivo ^1^H spectra and orange lines are denoised spectra Zoomed spectra from the red box are shown and artifact peaks and losses of signals are indicated with violet arrows, r, rank; MSE, mean squared error. Figure S4: Singular value decomposition of Hankel matrices H. The singular values (blue lines) and their log (orange lines) as a function of rank r. Singular values decrease exponentially with rank, with earlier singular values being much larger than later ones.
