# Supplementary material for: Signal-to-Noise Ratio Enhancement of Single-Voxel In Vivo 31P and 1H Magnetic Resonance Spectroscopy in Mice Brain Data Using Low-Rank Denoising
Source: Metabolites. 2022 Nov 29;12(12):1191. doi: 10.3390/metabo12121191 (PMC9782548; doi:10.3390/metabo12121191)
Supplement: Supplementary file 1 [file metabolites-12-01191-s001.zip › metabolites-2011761-supplementary.pdf]

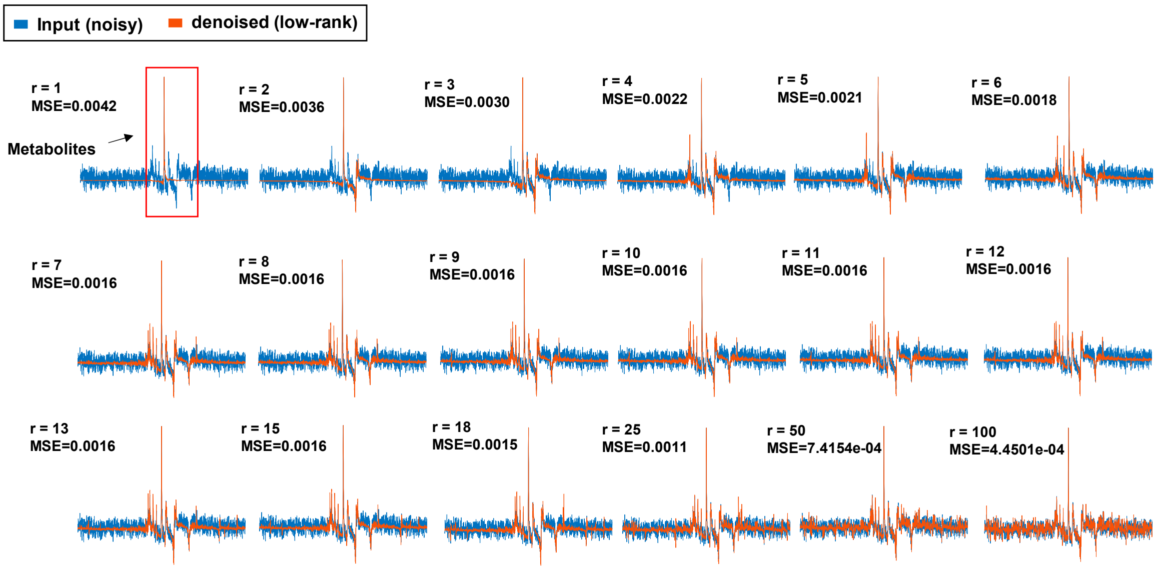

Figure S1. The low-rank denoising results of *in vivo* ^{31}P MRS normal mouse brain data with gradually increasing the number r. The blue lines are input noisy *in vivo* ^{31}P spectra and orange lines are denoised spectra, r, rank; MSE, mean squared error.

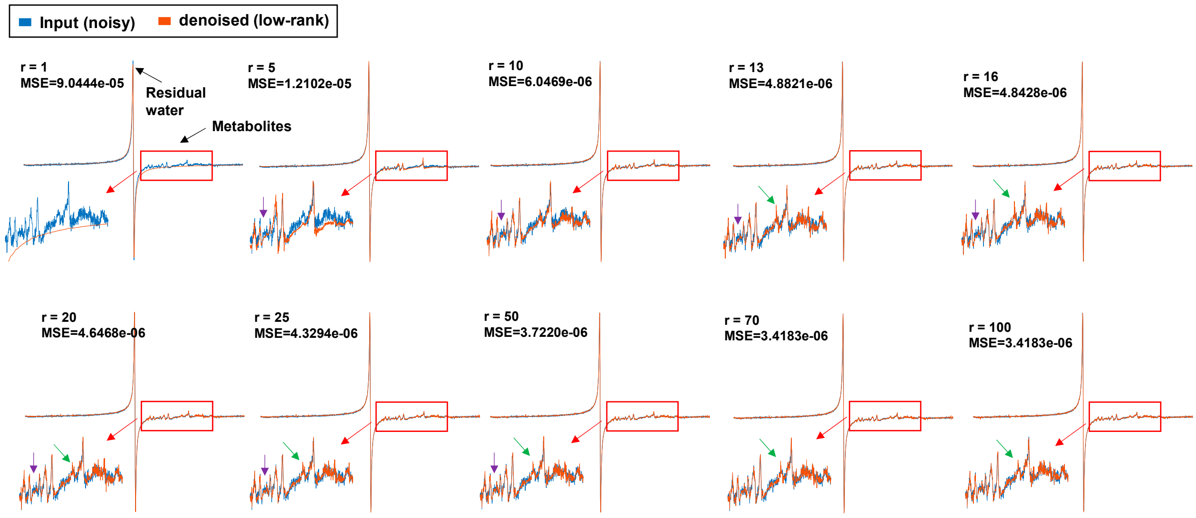

Figure S2. The low-rank denoising results of *in vivo* ^{1}H MRS normal mouse brain data with gradually increasing the number r. The blue lines are input noisy *in vivo* ^{1}H spectra and orange lines are denoised spectra Zoomed spectra from red box are shown and artifact peaks and loss of signals are indicated with green and violet arrows, r, rank; MSE, mean squared error.

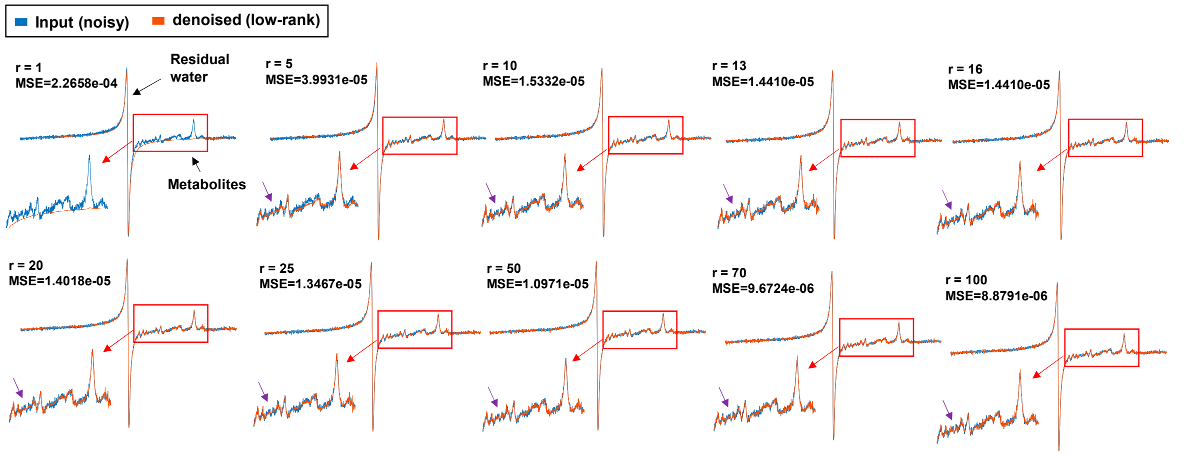

Figure S3. The low-rank denoising results of *in vivo* ^{1}H MRS stroke mouse brain data with gradually increasing the number r. The blue lines are input noisy *in vivo* ^{1}H spectra and orange lines are denoised spectra Zoomed spectra from red box are shown and artifact peaks and loss of signals are indicated with violet arrows, r, rank; MSE, mean squared error.

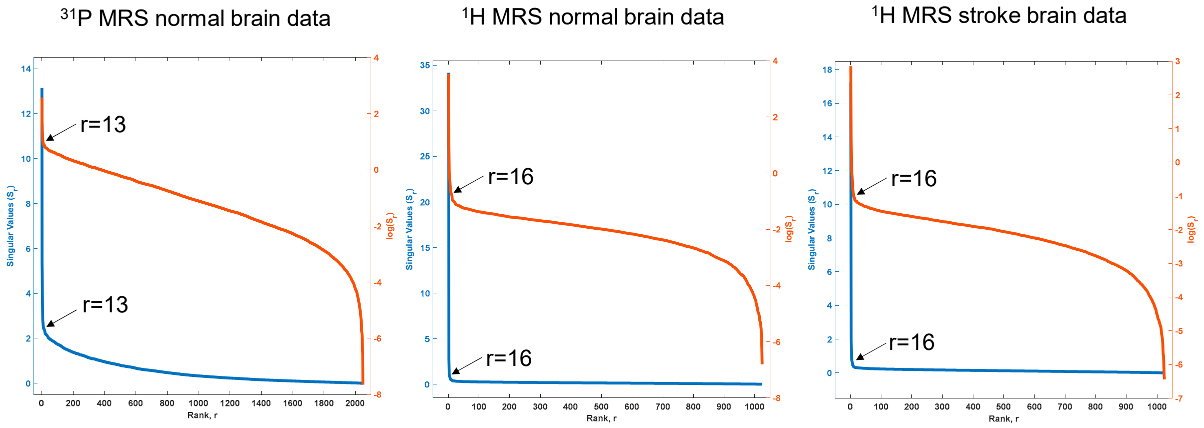

Figure S4. Singular value decomposition of Hankel matrices H. The singular values (blue lines) and their log (orange lines) as a function of rank r. Singular values decrease exponentially with rank, with earlier singular values being much larger than later ones.
